# Longitudinal study of leptin levels in chronic hemodialysis patients

**DOI:** 10.1186/1475-2891-10-68

**Published:** 2011-06-15

**Authors:** Ilia Beberashvili, Inna Sinuani, Ada Azar, Hila Yasur, Leonid Feldman, Zhan Averbukh, Joshua Weissgarten

**Affiliations:** 1 Nephrology Division, Assaf Harofeh Medical Center, Zerifin, Affiliated to Sackler Faculty of Medicine Tel Aviv University, Israel; 2 Nutrition Department, Assaf Harofeh Medical Center, Zerifin, Affiliated to Sackler Faculty of Medicine Tel Aviv University, Israel

**Keywords:** Leptin, Nutrition, Bioimpedance, Inflammation, Hemodialysis

## Abstract

**Background:**

The influence of serum leptin levels on nutritional status and survival in chronic hemodialysis patients remained to be elucidated. We conducted a prospective longitudinal study of leptin levels and nutritional parameters to determine whether changes of serum leptin levels modify nutritional status and survival in a cohort of prevalent hemodialysis patients.

**Methods:**

Leptin, dietary energy and protein intake, biochemical markers of nutrition and body composition (anthropometry and bioimpedance analysis) were measured at baseline and at 6, 12, 18 and 24 months following enrollment, in 101 prevalent hemodialysis patients (37% women) with a mean age of 64.6 ± 11.5 years. Observation of this cohort was continued over 2 additional years. Changes in repeated measures were evaluated, with adjustment for baseline differences in demographic and clinical parameters.

**Results:**

Significant reduction of leptin levels with time were observed (linear estimate: -2.5010 ± 0.57 ng/ml/2y; p < 0.001) with a more rapid decline in leptin levels in the highest leptin tertile in both unadjusted (p = 0.007) and fully adjusted (p = 0.047) models. A significant reduction in body composition parameters over time was observed, but was not influenced by leptin (leptin-by-time interactions were not significant). No significant associations were noted between leptin levels and changes in dietary protein or energy intake, or laboratory nutritional markers. Finally, cumulative incidences of survival were unaffected by the baseline serum leptin levels.

**Conclusions:**

Thus leptin levels reflect fat mass depots, rather than independently contributing to uremic anorexia or modifying nutritional status and/or survival in chronic hemodialysis patients. The importance of such information is high if leptin is contemplated as a potential therapeutic target in hemodialysis patients.

## Background

In recent years, the number of patients with end-stage renal disease (ESRD) has been increasing worldwide [1]. Depending in part upon the method used to evaluate nutritional status and the population studied, from 40 to 70 percent of patients with ESRD are malnourished [2,3] resulting in poor clinical outcomes [4]. Among the mechanisms responsible for malnutrition, leptin was believed to influence nutritional markers in patients with ESRD [5]. Leptin is a 16-kDa protein identified as the product of the obese gene; it is exclusively produced in adipocytes, and regulates food intake and energy expenditure in animal models [6]. Leptin decreases food intake by decreasing NPY (neuropeptide Y - one of the most potent stimulators of food intake) mRNA [7] and increasing alpha-MSH (alpha-melanocyte-stimulating hormone - an inhibitor of food intake) [8]. Besides linking adiposity and central nervous circuits to reduced appetite and enhanced energy expenditure in the general population [9], leptin has been shown to increase overall sympathetic nerve activity [10], facilitate glucose utilization and improve insulin sensitivity [11]. Furthermore, the prospective West of Scotland Coronary Prevention Study (WOSCOPS) reported that elevated leptin increases the relative risk of cardio-vascular disease in the general population independently of fat mass [12].

In general, serum leptin levels are significantly elevated in patients with renal failure, particularly when compared to age, gender and body mass index (BMI)-matched controls [13,14]. However, the role of hyperleptinemia in ESRD patients is somewhat unconventional. In contrast with its anorexogenic effects recognized in the general population [9] and even in experimental models of uremia (in subtotal nephrectomized and leptin receptor-deficient [db/db] mice) [15], leptin has not been reported to affect perceived appetite and nutrient intake in dialysis patients [16,17]. Although in some observational studies, increased serum leptin concentrations were observed in ESRD patients in parallel with loss of lean body mass [18,19] or with hypoalbuminemia and low protein intake [20], some others failed to find any correlation between hyperleptinemia and weight change [9] or lean mass [21] in this population. Moreover, several clinical studies suggested that leptin is a negative acute phase protein [22] and can serve as a marker of adequate nutritional status, rather than an appetite-reducing uremic toxin in hemodialysis patients [23-25]. Finally, the relationship between elevated serum leptin levels and clinical outcomes in ESRD has not been fully defined. In one small prospective cohort of hemodialysis patients, lower baseline serum leptin levels predicted mortality [26], but neither changes in leptin over time were measured, nor were leptin levels normalized to body fat mass in this study.

Thus, the influence of serum leptin levels on nutritional status and survival in chronic hemodialysis patients remained to be elucidated. In view of leptin's physiological role, information on effects of prolonged hyperleptinemia (independent of fat mass) on nutritional status of chronic hemodialysis patients, which may also impact on their survival, would be of interest. The aim of the present prospective longitudinal study was therefore to study longitudinal changes in serum leptin levels and to relate them to the changes in nutritional markers and survival in chronic hemodialysis patients.

## Methods

### Patients

**Figure 1:**
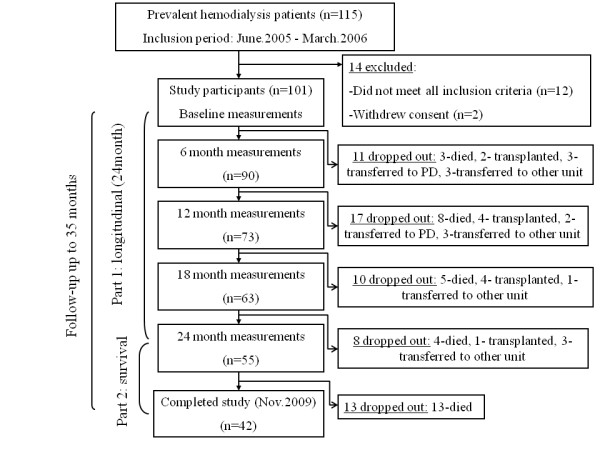
**Flow diagram of the study**.

This prospective observational study was approved by the Ethics Committee of Assaf Harofeh Medical Center (Zerifin, Affiliated to the Sackler Faculty of Medicine Tel Aviv University, Israel). Informed consent was obtained before any trial-related activities. Patients were eligible for entry when they had been on HD therapy for at least 3 months and were 18 years or older, with no clinically active cardio-vascular or infectious diseases on entry. We excluded patients with edema, pleural effusion or ascites at their initial assessment, as well as patients with malignant disease, liver cirrhosis, neuro-muscular diseases, amputations or any deformities of the body. Exclusion criteria at the entry of the study also included co-morbidity (auto-immune disease and/or acute infections) and/or medication (prednisone) that might interfere with plasma leptin concentrations. A flow chart of the study is presented in Figure 1. In total, 101 patients (64 men and 37 women) with a mean age of 64.6 ± 11.5 years, receiving maintenance hemodialysis treatment at our outpatient HD clinic, were included in the study. Of the patients studied, 52 were diabetic (all diabetic patients had type 2 diabetes). Study measurements were performed at baseline and at 6, 12, 18 and 24 months from enrollment. After the longitudinal measurements ended, we continued clinical observation on our cohort during 2 additional years. Thus, in total, the study period extended 35 ± 17 months. During this period, 33 patients (32.7%) died (the main causes of death were cardio-vascular [12 of 33 patients; 36.4%] and sepsis [12 of 33 patients; 36.4%]), 13 patients (12.9%) underwent kidney transplantation, 3 patients (3.0%) changed dialysis modality, and 10 patients (10.0%) transferred to other hemodialysis units. All patients underwent regular dialysis via their vascular access (81.2% of patients had arterio-venous fistula) 4-5 h three times per week at a blood flow rate of 250-300 ml/min. Bicarbonate dialysate (30 mEq/L) at a dialysis solution flow rate of 500 ml/min was used in all cases. All dialysis was performed with biocompatible dialyzer membrane with a surface area of 1.0-1.8 m^2^. The efficiency of the dialysis was assessed based on the delivered dose of dialysis (Kt/V urea) using a single-pool urea kinetic model (mean Kt/V was1.31 ± 0.23 in our population).

Information on vascular disease (cerebral vascular, peripheral vascular and heart disease) was obtained from a detailed medical history.

Most patients were required antihypertensive medications as well as other drugs commonly used in ESRD, such as phosphate and potassium binders, diuretics, and supplements of vitamins B, C, and D.

### Dietary intake

A continuous 3-day dietary history (which included a dialysis day, a weekend day and a non-dialysis day) was recorded on a self-completed food diary. Then, dietary energy and protein intake were calculated and normalized for adjusted body weight (ABW) by the following formula [27]:

SBW - standard body weight was determined by the National Healthand Nutrition Examination Survey II (NHANES II) population medians for age, sex, frame size, and stature [27].

Dietary protein intake was also estimated by the protein catabolic rate (PCR) calculation from the patient's urea generation rate by urea kinetics modeling [28]. Single-pool model urea kinetics was used to estimate the nPCR.

### Anthropometric measurements

BMI, triceps skinfold thickness (TSF), mid arm circumference (MAC) and calculated mid arm muscle circumference (MAMC) were measured as anthropometric variables. The BMI was calculated as dry weight in kilograms divided by the square of height in meters. TSF was measured with a conventional skinfold caliper using standard techniques. Mid arm circumference was measured with a plastic measuring tape. MAMC was estimated as follows:

### Body composition analysis

Body composition was determined by body impedance analysis (B.I.A. Nutriguard- M, Data-Input, Frankfurt, Germany). We used gel-based electrodes specifically developed for BIA measurements - Bianostic AT (Data-Input GmbH). On the day of blood collection, patients underwent BIA measurement at approximately 30 minutes postdialysis. BIA electrodes were placed on the same body side used for anthropometric measurements. The multi-frequency technique was used. FFM was calculated by using the approach of Kyle et al. validated by dual-energy x-ray absorptiometry on 343 healthy adult subjects [29]:

Fat mass and fat free mass were standardized by squared height (m^2^), and expressed in kg/m^2 ^as fat mass index (FMI) and fat free mass index (FFMI), respectively.

Phase angle (PA) describes the relationship between the two vector components of impedance [reactance and resistance] of the human body to an alternating electric current. PA has been shown to provide a BIA prognostic index of morbidity and mortality [30].

### Laboratory evaluation

Blood samples were taken in a non-fasting state before a midweek hemodialysis session. CBC, creatinine, urea, albumin, transferrin and total cholesterol were measured by routine laboratory methods. IL-6 and leptin were measured in plasma samples using commercially available enzyme-linked immunosorbent assay (ELISA) kits (R&D System, Minneapolis, MN, USA) according to the manufacturer's protocol. The mean minimal detectable level for IL-6 was 0.7 pg/ml, and 7.8 pg/ml for leptin. Intra-assay and inter-assay coefficients of variation for IL-6 were 4.2% and 6.4% respectively, and for leptin - 3.3% and 5.4% respectively.

### Statistical analysis

Data are expressed as mean ± standard deviation (SD), median and interquartile range (Q1 to Q3) for variables that did not follow a normal distribution, or frequencies, as noted.

To compare the means of continuous variables measured between sex-specific tertiles of leptin, one way ANOVA and analysis of covariance with adjustments (ANCOVA) were used. Categorical data are presented as percentages and were compared among groups by χ2 tests. Correlations between leptin and clinical and laboratory parameters were assessed using Spearman rank order correlation coefficients (because of the skewed distribution of leptin levels). Multivariate regression analysis was performed to obtain partial (adjusted) correlations (R^2^). Case-mix-adjusted models were controlled for age, gender, history of CV disease, presence or absence of diabetes mellitus, dialysis vintage and fat mass.

Repeated-measures analysis of variance was performed by using the MIXED model. Only patients with ≥ 2 study visits were included in the analyses. Base models were adjusted for age, sex, diabetes status, dialysis vintage, history of cardio-vascular diseases and fat mass. *F *Tests were used to assess the significance of the fixed effects, and *P *less than 0.05 was considered significant. To evaluate whether leptin influenced the trends in the various dependent variables, we included in each base model terms for individual "leptin-by-time" interactions.

Survival analyses were performed using the Kaplan-Meier survival curve and the Cox proportional hazard model. The univariate and multivariate Cox regression analyses are presented as (HR; CI).

All statistical analyses were performed using SPSS software, version 16.0 (SPSS Inc, Chicago, IL).

## Results

### Cross-sectional associations

The 101 prevalent HD patients participating in this study included 36.6% women; over half of the participants (51.5%) had diabetes mellitus (DM) and nearly the same proportion had a history of cardiovascular disease (46.9%), including myocardial infarction, coronary artery procedures such as angioplasty or surgery, previous cerebral-vascular accident, or peripheral vascular disease. Average age was 64.6 ± 11.5 years and median maintenance HD vintage was 31 months (Q1 to Q3, 15.5-51.5 months) at the study initiation. For HD patients at the start of the cohort, serum leptin averaged (mean ± SD) 37.3 ± 39.4 ng/ml (median, 16.5 ng/ml; Q1 to Q3, 5.60-71.4 ng/ml).

To examine the relationship between different levels of serum leptin and relevant demographic, clinical and laboratory measures, serum leptin levels were divided into three equal gender specific tertiles (data not shown). Diabetes proportion and age distribution were similar across the three groups. No statistically significant differences were evident between groups in the use of medications that may affect inflammatory markers such as statins, aspirin, or angiotensin-converting enzyme inhibitors (data not shown). As expected females, tended to have higher leptin levels than males (p = 0.0001). Body composition parameters measured by anthropometry and bioelectric impedance analysis (BIA) including phase angle (PA) were incrementally greater across increasing leptin tertiles even after adjustments for baseline demographic and clinical parameters (age, DM status, dialysis vintage and cardio-vascular disease in the past). However, these associations were attenuated after including of fat mass in multivariate analyses. No significant differences in any of the biochemical markers of nutrition, normalized protein nitrogen appearance (nPNA), or actual energy or protein intake normalized to adjusted body weight were found between the HD patient groups according to leptin tertiles.

At baseline, anthropometric measurements (body mass index [BMI], triceps skinfold thickness [TSF], mid arm circumference [MAC] and midarm circumference calculated [MAMC]) and BIA-derived parameters of body composition (fat mass index [FMI] and fat free mass index [FFMI]) correlated positively with serum leptin levels, in both unadjusted and adjusted (for age, DM status, dialysis vintage, and previous cardio-vascular disease) models. Thus, an increased level of leptin was associated with better nutritional status (Table 1). Associations between levels of serum leptin and the two laboratory nutritional markers (albumin and cholesterol) and PA derived by BIA were statistically significant, but these associations were attenuated after adjustments for demographic and clinical parameters including fat mass. No significant correlations were observed between serum leptin and nPNA or dietary energy (DEI) and protein intake (DPI) normalized to adjusted body weight in both sexes after adjustments for demographic and clinical parameters including fat mass.

**Table 1:** Unadjusted and multivariate adjusted Spearman's correlation coefficients of baseline serum leptin and nutritional clinical and laboratory parameters in the study population at baseline

	Raw		Adjusted^a^	
	R	P	R	P
**Dietary intake**				
Energy intake *(kcal/kg/d)*	-0.133	0.21	-0.170	0.17
Protein intake *(g/kg/d)*	-0.231	0.027	-0.177	0.15
nPNA	0.005	0.97	0.079	0.46
**Biochemical markers**				
Albumin *(g/dL)*	0.216	0.030	0.065	0.55
Creatinine *(mg/dL)*	-0.014	0.89	0.082	0.44
Cholesterol *(mg/dL)*	0.217	0.029	0.060	0.57
Transferrin *(mg/dL)*	0.161	0.11	0.056	0.61
IL-6 *(mcg/ml)*	-0.105	0.31	0.012	0.92
**Anthropometric measurements**				
BMI *(kg/m^2^)*	**0.758**	**< 0.001**	**-**	**-**
TSF *(mm)*	**0.696**	**< 0.001**	**0.319**	**0.008**
MAC *(cm)*	**0.751**	**< 0.001**	**0.491**	**< 0.001**
MAMC *(cm)*	**0.486**	**< 0.001**	**0.378**	**0.001**
**Bioimpedance analysis**				
FMI *(kg/m^2^)*	**0.862**	**< 0.001**	-	-
FFMI *(kg/m^2^)*	0.247	0.013	**0.296**	**0.014**
Phase angle *(°)*	**0.273**	**0.006**	0.128	0.297

Correlation coefficient values ≥ 0.25 appear in bold.

^a ^All case-mix-adjusted models include age, gender, DM status, dialysis vintage, CV disease in the past and fat mass.

Abbreviations: nPNA, normalized protein nitrogen appearance; IL-6, interleukin-6; BMI, body mass index; TSF, triceps skinfold thickness; MAC, mid-arm circumference; MAMC, mid-arm muscle circumference calculated; FMI, fat mass index; FFMI, fat-free mass index, DM, diabetes mellitus; CV, cardio-vascular.

### Longitudinal associationas

Linear mixed models were used to study the effects of longitudinal leptin changes on changes in nutritional parameters (slopes) over 24 months including fixed parameters such as age, gender, diabetes status, dialysis vintage, previous cardio-vascular events, and fat mass (Table 2). No significant changes in dietary intake or in any of the biochemical nutritional markers over time were found. In contrast, a significant reduction in body composition parameters (both those measured by anthropometry [BMI, TSF and MAMC] and by BIA [FMI, FFMI, PA]) over time was observed. However, leptin did not modulate the changes in outcome variables over time in our cohort (leptin-by-time interactions were insignificant).

**Table 2:** Regression coefficients with 95% Confidence Intervals for the effect of longitudinal leptin changes on nutritional parameters changes (slopes) during 24 months based on mixed-effects model with linear trends for variable and fixed parameters

	95% confidence interval					
	Estimate	Lower	Upper	F	P	P^a^
**Dietary intake**						
Δ DEI *(kcal/kg/d)*	-0.2285	-0.7376	0.2699	0.914	0.32	0.46
Δ DPI *(g/kg/d)*	-0.0119	-0.0334	0.0134	1.087	0.30	0.86
Δ nPNA	0.0020	-0.1572	0.0197	0.049	0.83	0.47
**Biochemical markers**						
Δ Albumin *(g/L)*	-0.2665	-0.4403	0.0928	2.371	0.13	0.37
Δ Transferrin *(mg/dL)*	-1.6025	-3.4108	0.2059	3.111	0.08	0.81
Δ Creatinine *(mg/dL)*	-0.0261	-0.1158	0.0635	0.336	0.56	0.52
Δ Cholesterol *(mg/dL)*	-2.9192	-4.8957	0.9428	1.676	0.24	0.32
Δ IL-6 *(mcg/ml)*	2.8850	-1.8151	3.9548	0.971	0.34	0.46
**EPO dose***(x10^2^u/kg/w)*	5.9104	-1.6371	13.4579	2.429	0.12	0.90
**Anthropometric measurements**						
Δ BMI *(kg/m^2^)*^b^	-0.1259	-0.2389	-0.0129	5.011	0.030	0.57
Δ TSF *(mm)*	-0.4376	-0.7181	-0.1571	10.100	0.003	0.09
ΔMAC *(cm)*	0.0409	-0.0945	0.1764	0.374	0.55	0.26
ΔMAMC *(cm)*	-0.2066	-0.3356	-0.0777	10.550	0.002	0.20
**Bioimpedance analysis**						
Δ FMI *(kg/m^2^) *^b^	-0.1467	-0.2697	-0.0211	5.512	0.021	0.76
Δ FFMI *(kg/m^2^)*	-0.1459	-0.2044	-0.0874	24.760	< 0.001	0.15
Δ Phase angle *(°)*	-0.1018	-0.1377	-0.0659	31.898	< 0.001	0.38

NOTE: All nutritional variables presented in Table were modeled separately as dependent variables, whereas independent variables included fixed factors (such as age, gender, diabetes status, dialysis vintage, past cardio-vascular disease and fat mass) and leptin as continuous variable. Terms for individual "leptin-by-time" interactions were included in each model. The model takes into account every measurements of leptin and presented nutritional variables in each time point for each patient separately. Presented regression coefficients demonstrate changes in outcome variables with time (24 months).

^a ^P for trend for leptin-by-time interactions.

^b ^FMI and BMI were adjusted only by age, gender, DM status, history of cardio-vascular disease and dialysis vintage.

Abbreviations: DEI, daily energy intake; DPI, daily protein intake; nPNA, normalized protein nitrogen appearance; IL-6, interleukin-6; EPO, erythropoietin; BMI, body mass index; TSF, triceps skinfold thickness; MAC, mid-arm circumference; MAMC, mid-arm muscle circumference calculated; FMI, fat mass index; FFMI, fat-free mass index; DM, diabetes mellitus; CV, cardio-vascular.

A mixed model including the fixed factors sex, age, DM status, dialysis vintage, history of CV disease and some of nutritional parameters predicted variability in leptin levels during the study presented in Table 3. We observed significant changes of leptin levels with time (linear estimate ± SE: -2.5010 ± 0.57 ng/ml/2y; p < 0.001). The effect of longitudinal changes of FMI on serum leptin variability over time was evident according to this model.

**Table 3:** Effects of longitudinal changes of nutritional parameters on changes (slopes) of leptin during 24 months based on mixed-effects model with linear trends for variable and fixed parameters

	95% confidence interval				
	Estimate	Lower	Upper	F	P
DEI *(kcal/kg/d)*	-0.0154	-0.3756	0.3448	0.007	0.93
Albumin *(g/L)*	0.0369	-0.7011	0.7750	0.010	0.92
IL-6 *(mcg/ml)*	0.0534	-0.0397	0.1465	1.289	0.26
EPO dose *(x10^2^u/kg/w)*	-0.0001	-0.0176	0.0173	0.0001	0.99
FMI *(kg/m^2^)*	5.4558	4.4808	6.4308	121.918	< 0.001
Age (years)	-0.2072	-0.5850	0.1706	1.187	0.28
Gender (men v women)	-11.7523	-21.2601	-2.2445	6.018	0.016
DM (yes v no)	-0.7272	-9.2410	7.7867	0.029	0.87
History of CV disease (yes v no)	-0.9497	-9.6537	7.7544	0.047	0.83
Dialysis vintage (months)	0.0341	-0.0849	0.1531	0.325	0.57
Time (months)	-2.5010	-3.5814	-1.4205	21.650	< 0.001

NOTE: The Table represents a simple Mixed-Model with leptin as dependent variable, and presented nutritional parameters and fixed factors (age, gender, DM status, dialysis vintage and history of CV disease) as independent variables. The model takes into account every measurements of leptin and presented nutritional variables in each time point for each patient separately.

Abbreviations: DEI, daily energy intake; IL-6, interleukin-6; EPO, erythropoietin; FMI, fat mass index; DM, diabetes mellitus; CV, cardio-vascular.

**Figure 2:**
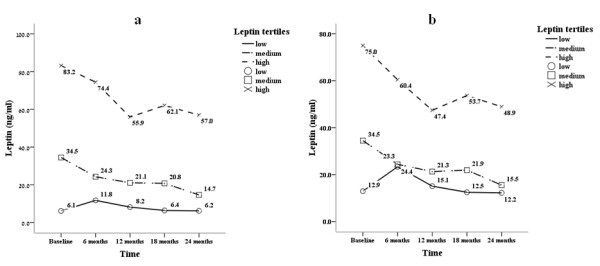
**Leptin levels decline over time in the study population**. Changes in estimated marginal means of serum leptin levels at baseline (month 0), month 6, month 12, month 18 and month 24 in the study population groups according to sex-specific tertiles* of baseline serum leptin: 1. Unadjusted model: P = 0.007 for leptin-by-time interaction; 2. Fully adjusted model (for age, gender, DM status, dialysis vintage, previous cardio-vascular morbidity and fat mass): P = 0.047 for leptin-by-time interaction. *Median (interquartile range) leptin (ng/ml) tertiles for men (n = 64) were 1.70 (1.2-2.4), 10.2 (7.6-13.8) and 38.3 (26.2-81.3) and for women (n = 37) were 15.8 (9.3-18.3), 68.0 (41.4-85.9) and 112.6 (109.2-116.0).

Of interest, a significant reduction over time was associated with a more rapid decline in leptin levels in the highest leptin tertile in both unadjusted (p = 0.007 for leptin-by-time interaction) and fully adjusted (p = 0.047 for leptin-by-time interaction) models (Figure 2).

### Survival analysis

**Figure 3:**
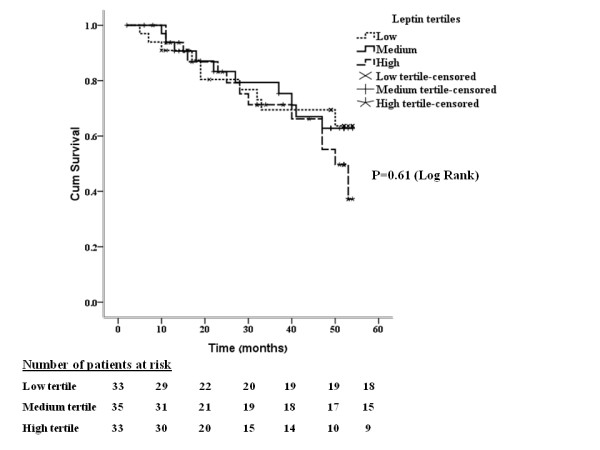
**Kaplan-Meier curves of surviving patients comparing subgroups of patients stratified by baseline serum leptin tertiles**.

During an average follow-up of 35 months (median, 40 months; Q1 to Q3, 17-52), 33 patients died: 11 (32%) in the low tertile group, 12 (35%) in the median tertile group and 10 (30%) in the high tertile group, with a median time to death of 25 months (Q1 to Q3, 14-40 months). Cumulative incidences of survival were unaffected by the baseline serum leptin levels (Figure 3). To further assess the possible association of baseline serum leptin level with cardio-vascular morbidity and mortality, cumulative hazards of all-cause death and first composite cardio-vascular event from Cox regression (after adjustment for baseline demographic parameters and fat mass) were calculated. First cardio-vascular event was defined as myocardial infarction (MI), requiring coronary artery procedures such as angioplasty or surgery, cerebral-vascular accident (CVA), or peripheral vascular disease (PVD), requiring angioplasty, bypass or amputation and diagnosed after the participant entered the study. No statistically significant differences in hazards between the different groups according leptin tertiles were observed. Additionally, no differences were found by Cox regression analysis in terms of survival probabilities from the analysis of all-cause mortality for each 10 ng/ml increase in baseline serum leptin (data not shown).

Thus, although chronic HD patients with higher baseline leptin had better nutritional status as evidenced by their significantly more favorable body composition parameters at the start of the study, this disparity did not appear to widen over the 24 months of follow-up; thus, leptin levels did not predict body composition parameter changes over time. Moreover, no survival advantage of the high leptin group was found in our observational cohort.

## Discussion

In the present study, we wished to determine whether changes of serum leptin levels are correlated with nutritional status over time in a cohort of prevalent hemodialysis patients. We observed that despite the strong and significant cross-sectional associations between serum leptin and body composition parameters at baseline, leptin levels did not reflect changes in nutritional status in our population.

Our study confirms several previous cross-sectional studies in which elevated serum leptin levels were demonstrated to be positively associated with several nutritional markers (laboratory or body composition parameters, mainly BMI and fat mass) in chronic HD patients [14,20,21,23-25]. HD patients treated with megestrole acetate (24), growth hormone [31] or high-calorie supplementation [32] demonstrated progressively increased serum leptin parallel to an improved nutritional state.

However, not all studies are consistent with positive associations between elevated levels of leptin and several nutritional parameters in ESRD patients. An inverse correlation was observed between leptin/fat mass and dietary intake, as well as with significantly lower lean tissue mass in chronic renal failure (23 patients) and ESRD patients receiving PD (24 patients) and HD (22 patients) therapy in study by Young et al [5]. In a cross-sectional study of 28 dialysis patients and 41 healthy control subjects, Johansen et al [20] showed that leptin levels were negatively correlated with albumin and PCR, suggesting a possible negative role of leptin in nutrition. Based on longitudinal observation, Stenvinkel et al [18] demonstrated that increases in serum leptin levels of 36 peritoneal dialysis patients were associated with a decrease in lean body mass. The discrepancies between these studies may be related to the population recruited, to their small sample size, and to their design; specifically, serum leptin levels were measured among patients with varying degrees of CRF, some of whom were undergoing maintenance hemodialysis and peritoneal dialysis, and in healthy controls [5,18,20]; many of these studies had a low number of study participants [5,18,20,23-25] and a majority of these studies were cross-sectional [5,20,23,25].

Leptin, a regulator of eating behavior, has been shown to be a major determinant of anorexia in uremic animals via signaling through the hypothalamic melanocortin system [15]. Subsequently, a recent experimental study by Cheung et al [33] showed that intraperitoneal administration of the melanocortin-4 receptor (MC4-R) antagonist NBI-12i stimulates food intake and weight gain in uremic mice. However, the effects of leptin in uremic patients are not unequivocal. Relevant to our findings, Bossola at al [17] demonstrated that serum leptin levels were not different in anorexic and in non-anorexic hemodialysis patients. Several other studies failed as well to demonstrate any role of hyperleptinemia on reduced dietary intake in ESRD patients receiving maintenance HD treatment [5,16,34]. Since hyperleptinemia is common in HD patients mainly due to impaired renal clearance [20], it seemed reasonable to assume that selective leptin resistance (such as occurs in obese humans [35]) is the responsible mechanism for attenuation of the negative effect of leptin on appetite, and accordingly, on dietary intake under conditions of continuous stimulation. The molecular mechanisms of leptin resistance, with a focus on the contribution of the intracellular tyrosine residues in the hypothalamic receptor of leptin (LEPRb) and their interaction with the negative feedback regulators, suppressor of cytokine signaling 1 and 3 (SOCS1 and SOCS3), are described in a recent study by Knobelspies et al [36].

Thus, although the cross-group comparisons confirm that hyperleptinemia is associated with better body composition parameters, no unequivocal associations in terms of biochemical data or dietary intake with serum leptin levels are evident in HD patients according to the available literature. Our study confirms these data.

Another finding of the present study was that 24 months of HD was associated with a significant reduction of plasma leptin levels, with a more rapid decline observed in patients with higher baseline leptin levels. Only limited information is available on the relationship between longitudinal serum leptin measurements and nutritional characteristics in chronic HD patients [18,32]. To the best of our knowledge, the present study is the first long-term longitudinal study to show the relationship between serum leptin level and nutritional status in ESRD patients receiving maintenance HD therapy. Several mechanisms may be involved in decrease of serum leptin levels over time in hemodialysis patients. These include, the increasing use of high flux and super-flux hemodialyzers [37], and/or use of alternative dialysis strategies such as hemodiafiltration [38]; recombinant human erythropoietin treatment (generally followed by a significant decline of leptinaemia in hemodialysed patients [39]); chronic inflammation (under the assumption that leptin, like albumin or transferrin, is a negative acute phase protein in chronic HD patients [22], although other studies [40] do not support this mechanism); metabolic acidosis, which can reduce the release of leptin from adipose tissue [41]; and finally, a decrease in fat mass leading to a decrease in the rate of leptin biosynthesis [14,18] (based on an insulin or a nutrient-sensing pathway regulating leptin gene expression in fat tissue [42]). Although patients in our facility were all treated using low flux hemodialysis, reduction of BMI and accordingly of fat mass over 24 months of the study may explain this longitudinal decrease of serum leptin levels in our cohort.

Of interest is the observation that adverse changes in body composition parameters occur over time, supporting the hypothesis that end-stage renal disease is associated with wasting as proposed in some [43,44] but not all [45] of the previous studies. The results of our study were consistent with those reported by Johansen et al [43] who did not find significant changes in any of the biochemical markers with time, but a significant reduction in phase angle over 1 year follow up was evident in prevalent HD patients. In parallel, together with reduced body composition parameters over time, we observed a longitudinal decline in serum leptin levels in our cohort. However, the lack of an association between serum leptin levels and future changes in body composition parameters (as exhibited by non-significant leptin-by-time interactions), suggests that the levels of leptin follow body composition (mainly fat mass) trajectory or its accompanying metabolic changes, rather than governing it.

Finally, serum leptin level did not appear as a useful predictor of all cause mortality or cardio-vascular morbidity in our study population during up to 4 years of observation. In this aspect, our findings support the results of earlier study by Tsai et al [46]. Although Scholze et al [26] showed a paradoxical reverse association between leptin level and clinical outcome in 71 chronic HD patients, our patients did not show such an association. The difference between our results and theirs might have been caused by differences in the cohorts. First, the population presented by Scholze et al [26] had a lower BMI at baseline than our patients, and no body composition parameters were measured. Further, the prevalence of diabetes mellitus was much greater in patients in the lower leptin group than in patients of the higher leptin group in the cohort of Scholze et al [26]. Indeed, HD patients with diabetes have a poor prognosis [4] that might be the cause of the lower survival rate in the lower leptin group in the above study [26]. While low levels of leptin may reflect a state of malnutrition in HD patients, proatherogenic effects of hyperleptinemia were linked to an adverse cardiovascular profile in general population [10,12]. We speculate that the lack of long-term benefits of hyperleptinemia in terms of survival of chronic HD patients could reflect the modulating influence of proatherogenic effects of high serum leptin levels. Finally, no relationships between serum leptin levels and previous cardio-vascular events were found by Diez et al [47] in a retrospective study on 82 dialysis patients. Zoccali et al [40] also did not show any difference in cardiovascular event-free survival in cohort of 192 hemodialysis patients after their stratification on the basis of the median value of plasma leptin.

Some limitations of the present study should be considered. First, this study is based on a relatively small sample size limiting detection of more subtle changes over time. Second, this study used only an observational approach, without manipulation of exposure factors, and therefore, no definitive cause-and-effect relationship can be derived for any of the risk factors analyzed. Dietary intake assessed by 3 day food records is another limitation of the study, as results can be subjective and incomplete, and can vary considerably from day to day as a result of dialysis treatment sessions and associated disturbances in food intake. Finally, significant circadian fluctuations of plasma leptin (regardless of the underlying degree of glucose tolerance, fasting, or diet) can certainly obscure some small but significant changes in plasma leptin [48] and may be a source of potential bias for longitudinal observation. Nevertheless, the present study has the advantage of providing long-term longitudinal data on the relationship between serum concentrations of leptin and nutritional parameters in prevalent HD patients.

## Conclusions

In conclusion, we found positive linear associations between serum leptin levels and body composition, whereas no significant relations in terms of biochemical data or dietary intake were evident in our cohort at baseline. Analyzing longitudinal data we concluded that any influence of serum leptin levels on nutritional status is limited to mirroring attained BMI and fat mass trajectory. Further, nutritional advantage at baseline of the study population with hyperleptinemia did not translate into long-term benefits in terms of survival. Taken together, our results suggest that in our cohort, leptin levels reflect fat mass depots, rather than independently contributing to uremic anorexia or modifying nutritional status and/or survival in chronic HD patients. These results are of particular importance if the use of subcutaneous injections of recombinant methionyl leptin [49] or leptin removal by super-flux polysulfone dialysers [37] is contemplated as a therapeutic intervention.

## List of abbreviations used

ESRD: end stage renal disease; HD: hemodialysis; DM: diabetes mellitus; CV: cardio-vascular; SBP: predialysis systolic blood pressure; DBP: predialysis diastolic blood pressure; EPO: erythropoietin; nPNA: normalized protein nitrogen appearance; nPCR: normalized protein catabolic rate; IL-6: interleukin-6; alpha-MSH: alpha-melanocyte-stimulating hormone; NPY: neuropeptide Y; DEI: daily energy intake; DPI: daily protein intake; ABW: adjusted body weight; SBW: standard body weight; BMI: body mass index; TSF: triceps skinfold thickness; MAC: mid-arm circumference; MAMC: mid-arm muscle circumference calculated; BIA: bioelectrical impedance analysis; FMI: fat mass index; FFMI: fat-free mass index; PA: phase angle.

## Competing interests

The authors declare that they have no competing interests.

## Authors' contributions

**IB **designed, organized and coordinated the study, managed data entry, contributed to data analysis and interpretation of data and wrote the manuscript. **IS **carried out the immunoassays, contributed to analyzing and interpretation of data and writing the manuscript. **AA **and **HY **carried out nutrition assessment (food intake analysis, body composition assessment), contributed to analyzing and interpretation of data and writing the manuscript. **LF **contributed to analyzing and interpretation of data. **ZA **and **JW **contributed to analyzing and interpretation of data and writing the manuscript. All authors read and approved the final manuscript.
